# Comparison of Conventional and Digital Workflows in the Fabrication of Fixed Prostheses: A Systematic Review

**DOI:** 10.7759/cureus.61764

**Published:** 2024-06-05

**Authors:** Mousumi Mahato, Sadananda Hota, Amulya Jain, Debanwita Dutta, Purnendu Bhushan, Anjana Raut

**Affiliations:** 1 Prosthodontics, Kalinga Institute of Medical Sciences, Kalinga Institute of Industrial Technology (KIIT) DU, Bhubaneswar, IND; 2 Prosthodontics, Kalinga Institute of Dental Sciences, Kalinga Institute of Industrial Technology (KIIT) DU, Bhubaneswar, IND; 3 Prosthodontics and Crown and Bridge, Kalinga Institute of Dental Sciences, Kalinga Institute of Industrial Technology (KIIT) DU, Bhubaneswar, IND

**Keywords:** precision of prosthesis, prosthodontic outcomes, success rate and feasibility, patient and operator satisfaction, long-term clinical performance, efficiency, accuracy, digital workflows, conventional workflows, fixed partial dentures

## Abstract

When considering dental restorations, the use of fixed partial dentures is one of the most widely accepted treatment options. In the past, fabrication was done using traditional techniques and the conventional workflow was by far the popular method; however, nowadays digital workflows are being used as a means to produce the prosthesis. This systematic review aims to compare the workflows by considering their respective qualities, such as precision, efficiency, cost-effectiveness, and clinical performance. A complete search has been carried out to incorporate any relevant studies published between the years 2012 and 2023 in databases such as Scopus, Web of Science, PubMed, ScienceDirect, and Cochrane Library. Two independent reviewers screened articles for inclusion and assessed the studies' methodological quality rating via the NIH Tool. A total of 22 relevant articles were reviewed after a systematic search strategy. The main outcome of the review was digital workflows were found to reduce working time, eliminate the selection of trays, minimize material consumption, and enhance patient comfort and acceptance. The studies also showed that digital workflows resulted in greater patient satisfaction and higher success rates than conventional workflows. Workflows for digital dentistry demonstrated to be better than traditional ones due to the cost-effectiveness, accuracy, and time optimization for the fabrication of fixed prostheses.

## Introduction and background

Background and rationale

The best possible dental prosthesis solutions and their continuous progress are due to the presence of prosthodontic methods. Traditionally, fixed partial denture (FPD) fabrication has remained closely associated with conventional workflows. While in the past years, new technologies have come into focus, digital technologies have become the new trend and show promise to be more smart and dynamic solutions. All clinicians need to understand this paradigm change and their comparative analysis is significant to facilitate evidence-based clinical decision-making [1-5].

Scope of the review

This study will focus on the systematic review inclusive of many different aspects in terms of conventional and digital workflow in the fabrication of fixed dental prostheses. With the scope spanning across a whole spectrum of aspects such as accuracy, marginal and internal fit level, efficiency, cost-effectiveness, patient-reported outcome measures, and longevity, it is therefore critical to ensure these innovations are standardized, accessible, and of high quality [6-8].

Objective

Moreover, the main purpose of this review is to evaluate literature sources and give a clear summary that addresses the benefits and disadvantages pertaining to the traditional and digital workflows during the procedure of fabrication of FPDs. We will narrowly concentrate on the thorough scrutiny of the available evidence and provide observations that should help the clinicians to match the most suitable approach to an individual patient considering both his/her needs and clinic requirements.

## Review

Materials and methods

**Figure 1:**
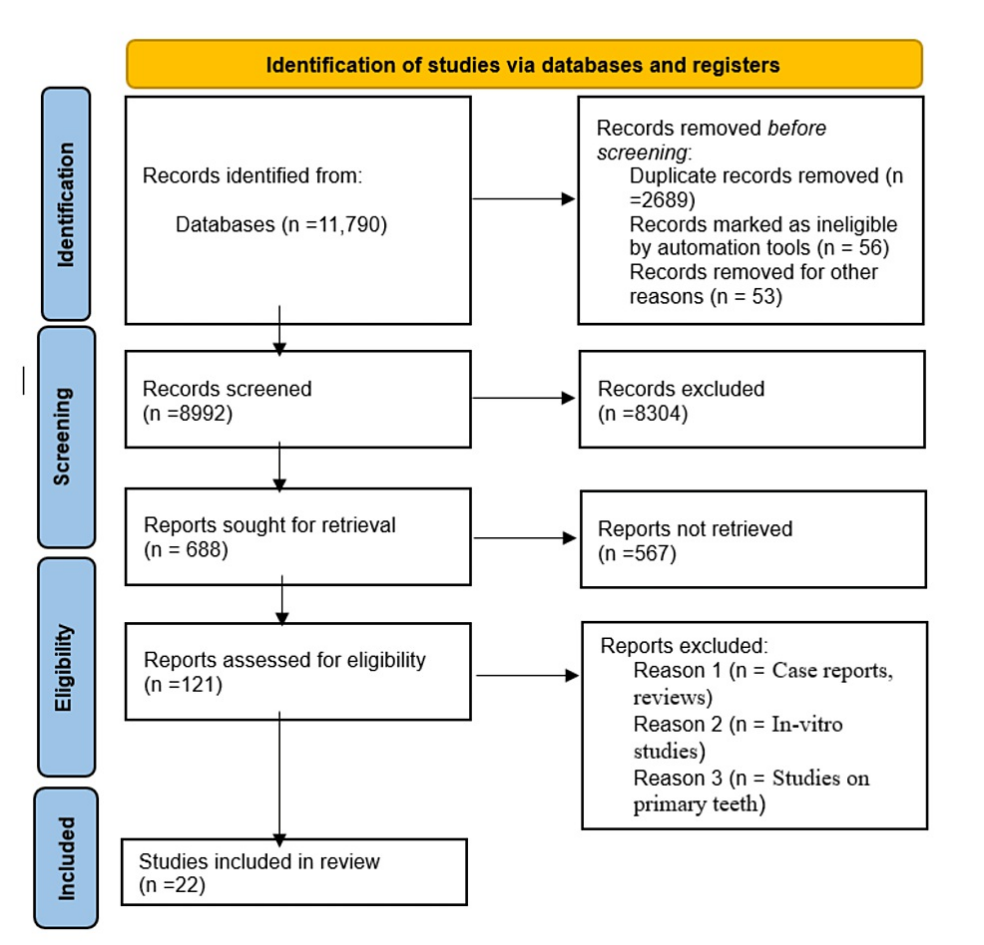
PRISMA Flow Diagram for Study Selection (2020) PRISMA: Preferred Reporting Items for Systematic Review and Meta-Analysis

This systematic review was conducted according to the Preferred Reporting Items for Systematic Review and Meta-Analysis (PRISMA) guideline (Figure 1). The protocol has been registered on PROSPERO (International Prospective Register of Systematic Reviews) (ID: CRD42023457500).

The PICO question was framed to identify and structure the fundamental components of systematic review: “Is a complete digital workflow with intraoral optical scanning (IOS) plus virtual design plus monolithic restoration for patients receiving prosthodontic treatments with tooth-supported or implant-supported fixed reconstructions comparable to conventional workflows with conventional impression and/or conventional casting and/or lost-wax-technique and/or framework and veneering in terms of feasibility in general or success-analysis including precision and accuracy assessment or economics or aesthetics or patient-centered factors?”

A MEDLINE (PubMed), Cochrane, Scopus, Science directory, and ScienceDirect search was conducted using the following search terms which were grouped into categories: Population: adult patients who require FPD or fixed dental prosthesis, Intervention: digital workflow in fabrication of FPD, Control: conventional workflow in fabrication of FPD and Outcome: success rate, accuracy, precision, time efficiency, cost efficiency, patient satisfaction, feasibility.

Search Strategy

A systematic search was performed in the following online databases: Scopus, Web of Science, PubMed, Science Direct, and Cochrane Library. Searches were conducted from 2012 to 2023; conference proceedings and abstracts were also accessed when possible. Electronic search strategies were customized to each database. Language was restricted to English in the search strategy. The Mesh (Medical Subject Headings) terms and keywords were paired together with the use of Boolean operators "AND" and "OR" to make keywords.

The following search strategy was used: ((“dental prosthesis”[All Fields]) OR (“crown”[All Fields]) OR (“dental crown”[All Fields]) OR (“implant supported prosthesis”[All Fields]) OR (“tooth supported prosthesis”[All fields]) OR (“fixed dental prosthesis”[All Fields]) OR (“FPD”[All Fields]) OR (“fixed reconstruction”[All Fields]) OR (“dental bridge”[All Fields]) OR (“implant crown”[All Fields]) OR (“implant restoration”[All Fields]) OR (“implant reconstruction”[All Fields]) OR (“fixed partial denture”[All Fields])) AND ((“computer aided design”[All Fields]) OR (“computer aided milling”[All Fields]) OR (“CAD-CAM”[All Fields]) OR (“digital workflow”[All Fields]) OR (“digital dentistry”[All Fields]) OR (“digital prosthodontics”[All Fields]) OR (“computerized dentistry”[All Fields]) OR (“intraoral scan”[All Fields]) OR (“digital scan body”[All Fields]) OR (“digital design”[All Fields]) OR (“cad/cam”[All Fields]) OR (“rapid prototyping”[All Fields]) OR (“monolithic”[All Fields]) OR (“full contour”[All Fields]) OR (“intraoral scanner”[All Fields])) AND ((“conventional workflow”[All Fields]) OR (“conventional impression”[All Fields]) OR (“impressions in dentistry”[All Fields]) OR (“hand layering”[All Fields]) OR (“veneering”[All Fields]) OR (“framework”[ All Fields]) OR (“wax pattern”[All Fields]) OR (“porcelain fused to metal”[All Fields]) OR (“PFM”[All Fields]) OR (“metal ceramic”[All Fields]) OR (“casting”[All Fields])) AND ((“survival”[All Fields]) OR (“survival rate”[All Fields]) OR (“success rate”[All Fields]) OR (“economics”[All Fields]) OR (”cost factor”[All Fields]) OR (“cost”[All Fields]) OR (“cost analysis”[All Fields]) OR (“esthetics”[All Fields]) OR (“patient satisfaction”[All Fields]) OR (“patient centered outcome”[All Fields]) OR (“feasibility”[All Fields]) OR (“study feasibility”[All Fields]))

Inclusion Criteria

Clinical trials and cross-sectional studies (RCT, non-RCT), studies on adult population with missing teeth and endodontically treated teeth, studies in which digital workflows were used for the fabrication of FPDs, studies in which conventional workflows were used for the fabrication of FPDs, articles with English language or other where English translation is possible, and articles from January 2012 to June 2023 were included.

Exclusion Criteria

Case reports, reviews, expert opinions, in-vitro studies, only abstracts, and studies on primary teeth/pediatric patients were excluded.

Systematic Search Strategy

Two independent reviewers (MM and SH) meticulously conducted a three-step selection process to identify relevant studies. The first stage was title scan where irrelevant studies were excluded based on a quick title review. The second stage was abstract and summary dive in which summaries and abstracts were examined to filter for studies based on the study type, digital intervention type, patient population size, and outcome variables assessed. The third stage was data extraction in which full texts of shortlisted studies were rigorously reviewed. Data relevant to study eligibility was extracted using a pre-defined form.

Data Extraction

The details of all the studies were collected as follows: year of publication, Name of the authors, study design, type of workflow or restoration, outcome assessed, and results. The data was collected by the reviewers separately and was converted in the form of a data extraction sheet, followed by compilation of the study results.

Study Risk of Bias and Quality Assessment

The quality and risk of bias of the included studies were assessed by the two independent reviewers. First, the duplicate sources were removed, and then study titles and abstracts were reviewed to ensure they are relevant. Following that, a full text review was done and the studies that fulfilled the inclusion criteria were included. Critical appraisal of randomized controlled trials (RCTs) and observational studies was done according to the NIH tool. The initial search resulted in 11790 articles. After removing irrelevant and duplicate articles, 121 full-text studies remained. These were then screened based on their titles and abstracts to assess eligibility according to the pre-defined inclusion/exclusion criteria. Ultimately, 22 articles were selected for in-depth qualitative analysis. Figure 1 describes the study selection process for this systematic review as per the PRISMA protocol [9].

Among these, 12 studies were RCTs, and the other 10 were non-randomized such as prospective cohort and observational studies.

Bias Assessment Within Individual Studies

Risk of bias in the RCT was divided considering the following criteria: the overall low risk of bias of the findings if all of the criteria were met; the unclear risk of bias if some criteria were met but at least one of them was only partially met; the high risk of bias if the findings lacked the fulfillment of at least one of the criteria. “High risk of bias” would imply “poor quality” and be assigned a grade. "Low risk of bias" refers to the grade of "good quality" rating. Four of the included RCTs would be considered to have a low-risk bias which makes them good quality while the other eight would be found to have an unclear risk bias which denotes that they are fair quality (Tables 1, 2).

**Table 1:** Quality Assessment in Randomized Studies (NIH) RCT: Randomized controlled trial; CD: cannot determine; NR: not reported.

	Sakornwimon et al. (2018) [10]	Mangano et al. (2018) [11]	Sailer et al. (2018) [12]	Rattanapanich et al. (2019) [13]	Mühlemann et al. (2019) [14]	Pan et al. (2019) [15]	Zhang et al. (2019) [16]	Kunavisarut et al. (2021) [17]	Capparé, (2021) [18]	Cheng et al. (2021) [19]	Ren et al. (2021) [20]	Seth et al. (2023) [21]
1. Was the study described as randomized, a randomized trial, a randomized clinical trial, or an RCT?	Yes	Yes	Yes	Yes	Yes	Yes	Yes	Yes	Yes	Yes	Yes	Yes
2. Was the method of randomization adequate (i.e., use of randomly generated assignment)?	Yes	NR	Yes	NR	CD	Yes	Yes	Yes	Yes	Yes	Yes	Yes
3. Was the treatment allocation concealed (so that assignments could not be predicted)?	NR	NR	yes	NR	NR	NR	NR	Yes	Yes	Yes	No	Yes
4. Were study participants and providers blinded to treatment group assignment?	NR	NR	NR	NR	NR	NR	No	Yes	NR	NR	No	NR
5. Were the people assessing the outcomes blinded to the participants' group assignments?	NR	NR	NR	NR	NR	NR	No	NR	NR	No	No	NR
6. Were the groups similar at baseline on important characteristics that could affect outcomes (e.g., demographics, risk factors, co-morbid conditions)?	Yes	NR	NR	Yes	NR	NR	NR	Yes	Yes	Yes	NR	Yes
7. Was the overall drop-out rate from the study at endpoint 20% or lower of the number allocated to treatment?	Yes	NR	NR	Yes	NR	NR	NR	Yes	Yes	NR	NR	NR
8. Was the differential drop-out rate (between treatment groups) at endpoint 15 percentage points or lower?	NR	NR	NR	NR	NR	NR	NR	Yes	CD	NR	NR	NR
9. Was there high adherence to the intervention protocols for each treatment group?	Yes	NR	NR	NR	Yes	NR	NR	Yes	NR	Yes	Yes	Yes
10. Were other interventions avoided or similar in the groups (e.g., similar background treatments)?	NR	Yes	NR	NR	Yes	NR	NR	No	NR	NR	Yes	Yes
11. Were outcomes assessed using valid and reliable measures, implemented consistently across all study participants?	NR	Yes	Yes	Yes	Yes	NR	NR	Yes	Yes	NR	Yes	Yes
12. Did the authors report that the sample size was sufficiently large to be able to detect a difference in the main outcome between groups with at least 80% power?	No	Yes	NR	NR	Yes	No	No	No	Yes	No	Yes	NR
13. Were outcomes reported or subgroups analyzed prespecified (i.e., identified before analyses were conducted)?	NR	NR	NR	NR	Yes	NR	NR	Yes	NR	NR	NR	NR
14. Were all randomized participants analyzed in the group to which they were originally assigned, i.e., did they use an intention-to-treat analysis?	NR	NR	NR	NR	Yes	NR	Yes	Yes	Yes	Yes	NR	NR
Quality Rating (Good, Fair, or Poor)	Fair	Fair	Fair	Fair	Good	Fair	Fair	Good	Good	Fair	Fair	Good

**Table 2:** Quality Assessment in Non-randomized Studies (NIH) NA: Not applicable; NR: not reported.

	Joda and Bragger (2014) [22]	Joda, Katsoulis, and Brägger (2015) [23]	Ferrini et al. (2018) [24]	Di Fiore et al. (2018) [25]	Yuce et al. (2019) [26]	Delize et al. (2019) [27]	De Angeliset al. (2020) [28]	Hashemi (2022) [29]	Edinger et al. (2023) [30]	Sanchez-Lara et al. (2023) [31]
1. Was the research question or objective in this paper clearly stated?	Yes	Yes	Yes	Yes	Yes	Yes	Yes	Yes	Yes	Yes
2. Was the study population clearly specified and defined?	Yes	Yes	NR	Yes	NR	Yes	Yes	Yes	Yes	Yes
3. Was the participation rate of eligible persons at least 50%?	Yes	Yes	NR	NR	NR	Yes	Yes	Yes	Yes	NR
4. Were all the subjects selected or recruited from the same or similar populations (including the same time period)? Were inclusion and exclusion criteria for being in the study prespecified and applied uniformly to all participants?	Yes	Yes	NR	Yes	NR	Yes	Yes	Yes	Yes	Yes
5. Was a sample size justification, power description, or variance and effect estimates provided?	No	No	NR	No	No	No	No	Yes	No	Yes
6. For the analyses in this paper, were the exposure(s) of interest measured prior to the outcome(s) being measured?	No	No	NR	NR	NR	No	Yes	No	No	Yes
7. Was the timeframe sufficient so that one could reasonably expect to see an association between exposure and outcome if it existed?	Yes	Yes	No	Yes	NR	No	Yes	Yes	NR	Yes
8. For exposures that can vary in amount or level, did the study examine different levels of the exposure as related to the outcome (e.g., categories of exposure, or exposure measured as continuous variable)?	Yes	Yes	Yes	NR	Yes	Yes	Yes	Yes	Yes	Yes
9. Were the exposure measures (independent variables) clearly defined, valid, reliable, and implemented consistently across all study participants?	Yes	Yes	NR	NR	Yes	Yes	Yes	Yes	No	Yes
10. Was the exposure(s) assessed more than once over time?	Yes	No	No	NA	Yes	No	No	Yes	Yes	No
11. Were the outcome measures (dependent variables) clearly defined, valid, reliable, and implemented consistently across all study participants?	Yes	Yes	NR	Yes	Yes	Yes	Yes	Yes	NR	Yes
12. Were the outcome assessors blinded to the exposure status of participants?	No	No	NR	NR	NR	No	No	No	No	Yes
13. Was loss to follow-up after baseline 20% or less?	Yes	Yes	NR	NA	NR	Yes	Yes	Yes	NR	NA
14. Were key potential confounding variables measured and adjusted statistically for their impact on the relationship between exposure(s) and outcome(s)?	Yes	Yes	NR	NR	Yes	Yes	No	Yes	NR	NR
Quality Rating (Good, Fair, or Poor)	Fair	Fair	Fair	Fair	Fair	Fair	Fair	Good	Fair	Good

The NIH tool was employed to evaluate the quality of the included studies [32]. Two studies scored a low risk of bias translating to good quality and eight studies scored an unclear risk of bias translating to fair quality (Tables 1, 2). Results of individual studies are shown in Table 3.

**Table 3:** Results of Individual Studies RCT: Randomized controlled trial; CAD/CAM: computer-aided-design and computer-aided-manufacturing

No.	Year	Author	Study design	Type of workflow or restoration	Outcome	Results
1.	2014	Joda and Bragger [22]	Crossover clinical study	N = 20, 2 x 20 implant crowns and treated with patient-specific titanium abutments and digitally designed ceramic superstructures as well as with standard titanium abutments and gold crown castings each.	Treatment costs	The digital workflow had a significantly lower direct treatment cost
2.	2015	Joda, Katsoulis, and Brägger [23]	Prospective cohort study	N = 20, cross-over design for single-tooth, posterior sites, each with a custom titanium abutment and CAD/CAM-zirconia-suprastructure and a standardized titanium abutment with PFM-crown.	The mean clinical adjustment time turned out to be distinctly lower in the digital workflow relative to that of the traditional one.	Time efficiency: best performance was shown by digital workflow. Cost efficiency: Digital pathways had significantly lower production costs compared to the mixed workflows. Patient satisfaction: No significant differences. The mean total clinical adjustment time did not differ significantly for the three groups.
3.	2018	Mangano et al. [11]	RCT	N= 50, 25 per group. Single implant restoration. 1^st^ group - monolithic zirconia crown, digital workflow, 2^nd^ group - metal ceramic crown, conventional workflow	Time, cost, success, complications, peri-implant bone loss, patient satisfaction	Cost efficiency: Digital procedure showed better cost efficiency Peri-implant marginal bone loss: No statistically significant differences. Patient satisfaction: Comfort during the impression procedure was deemed better in the digital methodology. Time efficiency: Digital workflow proved to be better.
4.	2018	Ferrini et al. [24]	Prospective clinical study	N= 24, posterior maxilla, immediately loaded tilted implants, 3 or 4- unit screw-retained prostheses Control group – definite traditional impressions. Test group – digital impressions	Evaluated implant success by examining framework-implant connection, survival rates, bone level changes, over 36 months.	No statistically significant differences when considering marginal bone loss.
5.	2018	Di Fiore et al. [25]		N= 10, posterior single implant. 1 group digital workflow, other conventional workflow	Operating time, clinical adjusting time, patient preference, self-perception of the esthetic outcome.	The mean operating time for the DW crowns was less than CW. The mean total adjustment times of CW was more than DW. The mean score regarding self-perception of the esthetic outcome was also more for DW.
6.	2018	Sailer et al. [12]	RCT	N= 10, 3-unit FPD. 3 intraoral digital scanners and respective workflows ([Lava], [iTero], Cerec Bluecam; Dentsply Sirona [Cerec]) were compared) with the conventional workflow using polyether (Permadyne; 3M)	Time efficiency. Participant and clinician perceptions were rated using visual analog scales.	Precision: No significant difference in marginal fit between workflows. However, conventional crowns showed a better fit in occlusal areas. Chairside milling resulted in a less favorable crown fit compared to centralized milling. Time Efficiency: Conventional methods were faster for both crown fabrication and finalization (group K vs. groups L, CiL, and CiD) compared to CAD/CAM. However, digital design was significantly quicker than conventional methods.Finalization of crowns took significantly longer in the fully chairside digital group (CiD) compared to all others.
7.	2018	Sakornwimon et al. [10]	RCT	Tooth-borne crowns, ZrO2 crowns 1^st^ group: digital workflow. 2^nd^ group : conventional workflow. 16 patients 32 crowns	Precision Self-perception and overall preference	Precision: No significant differences in clinical marginal fit. Patient satisfaction: 15 of the 16 patients preferred digital over conventional impressions.
8.	2019	Yuce et al. [26]	Clinical trial	30 heat-pressed and 31 CAD/CAM porcelain laminate veneers. Silicone replicas of each veneer were obtained and used to measure the adaptations. 12 patients.	Marginal and internal adaptation - clinical performance	The mean marginal and internal adaptation values of heat-pressed and CAD/CAM veneers were not statistically different . All veneers were rated 100% satisfactory during the two-year period.
9.	2019	Pan et al. [15]	RCT	Implant-supported crowns: ZrO2 crowns 1^st^ group; digital workflow (Trios by 3Shape). 2^nd^ group: conventional workflow (Impregum Penta). 40 patients 80 crowns	- Precision -Time efficiency	Precision: no statistically significant differences regarding interproximal and occlusal contact. Time efficiency: significantly lesser time for digital workflow. Model-free digital workflow took significant less laboratory time. Mean clinical chairside adjustment time showed no significant differences.
10.	2019	Zhang et al. [16]	RCT	Implant-supported crowns 1^st^ group: Monolithic lithium disilicate crowns using digital workflow. 2^nd^ group: ZrO2 framework followed by ceramic veneering. 33 participants, 1 crown each	-Precision -Time efficiency	Precision: digital group showed fewer adjustments and better fabricating accuracy compared with the conventional group. Time efficiency: Complete digital workflow showed significantly shorter clinical and laboratory times.
11.	2019	Delize et al. [27]	Non-RCT	Screw retained single implant crowns. N= 34, including within‐patient comparison of two workflows. 1 group - monolithic crown from complete digital workflow. Control group - conventional workflow, veneered zirconia	Assessment of Prosthodontic outcomes such as occlusion, PROMs, esthetic results using the white esthetic score (WES)	No significant differences in the occlusion and interproximal contacts between the two workflows whereas the global WES was significantly higher in the conventional group. Patient satisfaction scores, analyzed using visual analog scales (VAS), were significantly better for the digital group than for conventional impressions. The conventional group showed significantly higher value in the patients’ perception of the esthetic outcomes.
12.	2019	Rattanapanich et al. [13]	RCT	n=25: immediate loading implant with digital technique n=25: conventional loading implant treatment.	- Clinical and radiographic outcome were evaluated over a period 12 months - Patient satisfaction.	No statistically significant differences in success rate and marginal bone level. In patient satisfaction, the only statistic significant difference was in the question related to implant prosthetic function in favour of the conventional group, whereas there was no difference displayed in the question regarding speaking, cleansing, price, and expectation.
13.	2019	Mühlemann et al. [14]	RCT	N = 10, 3 unit FPD, 4 groups. Group L – lava, Group iT – institute Straumann, Group C – Cerec and Group K – conventional workflow	-Fabrication time -Chairside time	Precision: No statistically significant differences amongst the groups; conventionally fabricated crowns showed better marginal adaptation Time efficiency: No statistical differences in the total clinical treatment time
14.	2020	De Angelis et al. [28]	Multicenter retrospective clinical study	Implant supported singles crowns, 3 unit FPD. N= 64; fully digital approach N = 58; conventional protocol.	patient and operator centered outcomes - 2 visual analog scale (VAS) questionnaires	The VAS questionnaire for patients showed better results for the digital workflow regarding anxiety, convenience, taste, nausea sensation, pain and breathing difficulties. The VAS questionnaire for operators showed better scores for the digital approach in relation to convenience, anxiety and difficulties of the impression procedure. A significant reduced mean time and reduced number of visits were recorded for the digital workflow.
15.	2021	Capparé et al. [18]	RCT	N= 50, single implants. 25 patients; immediate-loading protocol using the digital workflow 25 patients; conventional workflow.	Clinical and radiographic factors were assessed at the time of implant insertion and over a period of 12 months. Pink Esthetic Score (PES) and patient satisfaction were also assessed.	Esthetics: No significant difference in the mean total PES. Patient satisfaction: preference for digital workflow was more than conventional workflow. Peri-implant bone loss: no statistically significant differences after a 12 month follow up. Time efficiency: digital workflow was considered more time-efficient.
16.	2021	Cheng et al. [19]	RCT	Tooth-supported crowns, 40 patients, 40 PMMA interim crowns 1st: digital workflow, experienced clinicians. 2^nd^ : digital workflow, less experienced clinicians. 3^rd^ : conventional workflow, experienced clinicians. 4^th^ : conventional workflow, less experienced clinicians.	Precision Time efficiency	Precision: Digital workflow – better precision occlusally, no significant differences in marginal fit, proximal contact, crown morphology amongst the groups. Time efficiency: Mean laboratory as well as clinical time was significantly less for digital workflow and Significant difference between experienced and less-experienced clinicians in terms of clinical time with the conventional workflow was found. When considering less-experienced clinicians, overall time was reduced by using the digital workflow.
17.	2021	Ren et al. [20]	RCT	Implant-supported crowns, 40 patients, 40 provisional crowns N = 20: IOS and digital workflow. N = 20 : conventional impressions and hybrid workflow.	Precision Time efficiency	Precision: Significant difference was found in crown adjustments; Precision on the occlusal surface was found to be better in the complete digital workflow. Time efficiency: Significantly shorter clinical and laboratory times in complete digital workflows.
18.	2021	Kunavisarut et al. [17]	RCT	N = 20, implant supported single crowns. Each group was then equally divided into two: lithium disilicate and polymer-infiltrated ceramic networks groups	Patient satisfaction was assessed (patient reported outcome measures)	Patient satisfaction: IOS showed significantly less taste irritation than the conventional impression technique. Similar results for both impression procedures regarding duration of procedures, comfort, level of anxiety, nausea, and pain No significant differences between Lithium disilicate and PICN crowns
19.	2022	Hashemi et al. [29]	Prospective Crossover Clinical Trial	N = 10, 3-unit implant supported FDPs in the posterior mandible. Frameworks fabricated using zirconia and cobalt–chromium, respectively for each patient	Comparison of Digital and Conventional Impressions, fitness and passivity, occlusion, aesthetics and treatment duration	Accuracy and passivity: No significant differences. Esthetic: No significant difference in the mean VAS score. Time efficiency: No significant difference in the mean clinical time. Mean laboratory time was shorter for the digital workflow. Occlusal adjustment time – no significant differences. Total fabrication time - significantly shorter for the digital workflow.
20.	2023	Seth et al. [21]	Randomized crossover study	N = 40, single tooth implant. Patients underwent both digital and conventional procedures. Only the designated impression or scan was sent to the dental laboratory to be processed.	Questionnaire was prepared regarding preferred technique. profile (OHIP-14) questionnaire was given before and after treatment. The Copenhagen Index Score (CIS) was used to assess the esthetic and technical quality.	Participants and students both preferred the digital technique. The CIS showed no significant difference and the OHIP-14 scores showed a significant drop.
21.	2023	Edinger et al. [30]	Clinical trial	N = 40 for each group, implant supported single crown. In 1 group, an index was taken immediately after implant placement using composite resin for fabrication of final crowns. For the other group, intraoral intraoperative scans were done during primary surgery. The custom-fabricated screw-retained crowns were placed during second-stage surgery.	Modified pink esthetic score (PES) was assessed. The functional implant prosthetic score (FIPS) was measured.	No significant differences in the FIPS and PES. However, the conventional workflow showed better values for the papillae.
22.	2023	Sanchez-Lara et al. [31]	Prospective clinical study	N = 22, for each patient, a final impression was made using polyether and three different intraoral scanners: CEREC Omnicam, Planmeca Planscan and True Definition. For the Polyether group, lithium disilicate ceramic was used and for the other three groups, dedicated CAD–CAM systems and materials were used.	Digital superimposition software was used to assess the marginal and internal differences between the crowns and tooth preparation at various locations.	Vertical margin discrepancy - greater than 120 μm in crowns - CAD–CAM systems and below 100 μm -conventional technique. Horizontal marginal discrepancy : only CEREC CAD–CAM was below 100 μm. Internal discrepancy was less for crowns fabricated with an analog workflow.

Studies reveal that digital workflows in prosthetic treatment may improve efficiency and patient comfort. Several studies (Joda et al. [22], Mühlemann et al. [14], etc.) showed evidently reduced chair time compared to conventional methods. Digital techniques were also linked to better patient satisfaction (Mangano et al. [11] and Kunavisarut et al. [17]) and improved clinical outcomes (Zhang et al. [16], Cheng et al. [19], and Ren et al. [20]). While clinicians preferred traditional impressions (Sailer et al. [12]), patients favored digital scans (Sakornwimon et al. [10]) and outcomes (Edinger et al. [30]). Overall, digital workflows appear to offer advantages in efficiency, patient experience, and clinical results.

Discussion

The studies compared the digital workflow and conventional workflow based on different factors. 

Time Efficiency

Zhang et al. [16] and Capparé et al. [18] found that complete digital workflows significantly reduced the clinical and laboratory times. The median adjustment count was also lower in the digital group. Cheng et al. [19] and Mangano et al. [11], found that the mean laboratory and clinical time was lesser for the digital workflow. Ren et al. [20] found that the mean chair-side time and laboratory time were significantly shorter in the test group (provisional + Zr crowns IOS and digital workflow) compared to the control group (provisional + Zr crowns using conventional impressions and hybrid workflow). Pan et al. [15] concluded that the digital workflow was more time-efficient compared to the conventional workflow. The operating and adjusting times were lesser for the digital workflow according to Fiore et al. [25] and Sailer et al. [12]. In summary, the studies consistently showed that digital workflows were associated with improved time efficiency compared to conventional workflows, leading to shorter laboratory and clinical times among different types of dental prostheses.

Cost Efficiency

The research of Joda et al. [23] suggested that the digital workflow resulted in considerably lower production costs than the mixed and digital workflows. Additionally, a research study by Joda and Bragger [22] and Mangano et al. [11] showed that the direct treatment costs were statistically lower for the digital workflow compared to the traditional workflow. According to the study by Zhang et al. [16], the cost efficiency was evaluated using production costs, and it was revealed that costs were lower with the digital version. Nonetheless, it is necessary to take into account the fact that the exact price difference and the amount of time and expenses saved may be different when determining other workflows and materials used in other clinical settings. In order to determine whether digital workflows are more cost-efficient than traditional methods for dental restoration, further research or a specific cost analysis would be needed. 

Patient Satisfaction

The study by Delize et al. [27] reported a statistically significant higher visual analog scale (VAS) satisfaction scores for IOS when compared with traditional impressions. It is clear that patients reported less taste disturbance with IOS and compared to conventional impressions. On the other hand, the patients’ aesthetic outcomes in the control group that used the conventional workflow with veneered zirconia (control group) received a high value in this study. Patient satisfaction was consistent regarding the procedure duration, comfort level, anxiety level, and nausea according to Kunavisarut et al. [17], but taste irritation was significantly less in the case of IOS than conventional impressions. Similarly, in the study by De Angelis [28], patients demonstrated satisfaction results among the digital workflow that were evaluated on the convenience, anxiety, taste, nausea, pain, and breathing problems on the VAS questionnaire. Another important finding of the study is that the mean time for the digital workflow had improved significantly. The result of the study by Rattanapanich et al. [13] showed that there was no difference in the patient satisfaction regarding implant loading approach of digital workflow compared to traditional implant therapy at the one-year follow-up. According to Capparé et al. [18], patients preferred the digital workflow over the conventional workflow.

Operator Satisfaction

The assessment of operator satisfaction was performed in the study by De Angelis et al. [28] by using VAS questionnaires. The questionnaire obtained better results with the digital workflow regarding anxiety relief, convenience in the use, satisfaction with taste, discomfort caused by nausea, relief of chest pain, and difficulty breathing. The digital protocol's scores were better than those for the conventional procedure in terms of anxiety incidences, convenience, uncomfortable impression procedures, and workflow. To elaborate further, the digital method led to the reduction in the meantime and the number of visits, which stated a higher satisfaction degree among operators with the digital manner. The work carried out by Sven Mühlemann et al. [14] reported that statistical differences did not occur during the accuracy assessment for both groups while there was a trend towards a better marginal adaptation for the traditionally fabricated crowns. However, the study concluded that the overall treatment time did not bring about any constructional difference. According to Yuce et al. [26], the mean marginal and internal adaptation values of heat-pressed and CAD/CAM veneers were not statistically different. According to Seth et al. [21], both participants and students preferred the digital approach. In general, the studies concluded that user satisfaction was higher in digital than in conventional system and digital workflow correlated with shorter duration of time and higher usability for operators. Moreover, the digital approach would be proven to be more time-efficient in some cases, resulting in an operator satisfaction increase.

Accuracy and Fit of Prostheses

Clinical marginal fit: In the study of the clinical marginal fit of ZrO_2_ crowns, the authors did not find a difference between the use of digital or conventional impressions (p>0.05) (Sakornwimon et al. [10]). Research by Zhang et al. [16] didn't find any differences in marginal fit, proximal contact and crown morphology for the digital and conventional workgroups (p> 0. 5). The test group in the study by Zhang et al. [16] had less adjustment and greater accuracy than the control group. The study by Pan et al. [15] proved to be the same regarding the contact interproximally and occlusal contact for digital and conventional fabrication processes. The accuracy of digital workflows, with special emphasis on the precision of a clinical marginal fit, patient's suitability, adjustments, fabricating precision, and contact quality seems at the same level as conventional ones. The results might have the specific outcome, yet they may be different for different restoration types used. This demonstrates that the accuracy or the quality of the outcomes was similar with these two workflows.

Prosthodontic Outcomes

A study by Delize et al. [27] found comparable results for occlusion and interproximal contacts for two workflows but showed significantly higher aesthetic results and patient satisfaction scores for the conventional workflow. Edinger et al. [30] measured the pink esthetic score and functional implant prosthetic score and there were found to be no significant differences between the two workflows. A 2020 study by De Angelis et al. [28] showed that the results were assessed using VAS questionnaires for both the patients and operators. According to the results, the digital workflow was better in terms of convenience, anxiety, taste, nausea sensation, pain, breathing difficulties and duration of the procedure, all suggesting the likelihood of digital workflow being beneficial for the patients and the operator. In addition, a clinical trial was conducted in a controlled environment through randomization in 2019 by Rattanapanich et al. [13], which compared immediate loading implant treatment by the digital approach with conventional loading implant treatment. The analysis consisted of the evaluation of clinical results, radiographic bone level and patient satisfaction. The findings of the study were similar, in which there was no statistically significant difference in the implant success rate and marginal bone level, whether the digital workflow or the conventional implant treatment was used. While on the other hand, there was a significant difference between the two groups in respect of their degree of satisfaction towards implant prosthetic function in favor of the conventional group. The 2023 prospective clinical study by Sanchez-Lara et al. [31] determined marginal and internal gaps between crowns and the prepared tooth. The outcomes were that the marginal dimension discrepancies in the medial and lateral directions differed among various protocols, with the ones made with CAD-CAM technology showing a variation in the marginal discrepancy across different systems of scanning and fabrication methods. This suggests that the digital workflow has potential to take an edge on FPD achievement by way of specific fit or precision compared to traditional methods, again in relation to the specific approaches and technologies under consideration.

Success Rate and Feasibility

In the study by Joda et al. [23], it was found that laboratory cross-mounting was feasible for three reconstruction pairings, revealing a 15% vice versa transfer success rate for the digital workflow. Moreover, with the digital workflow, the average clinical adjustment time was substantially less when compared with the procedures performed using the conventional method. In a cross-over clinical trial conducted in 2022 by Hashemi et al. [29], the effectiveness of traditional impressions and digital impressions was compared with respect to various factors such as proper fit, occlusion, aesthetics, and treatment duration. Marginal bone-loss was not statistically different between the two workflows according to Ferrini et al. [24]. In summary, the investigations demonstrate that the application of digital workflows offers advantages relative to FPD fabrication, for instance, reduced discomfort for patients and staff and greater precision made possible ultimately not only by faster production but also potential cost cutting.

## Conclusions

This collection includes studies that show a comparison between the digital and conventional workflow in dental implant and prosthodontic treatments that includes different aspects such as treatment outcomes, patient satisfaction, precision, time efficiency, and cost-effectiveness. In all these studies, the results obtained point to several key findings that are highlighted. These benefits involve considerable time and cost reduction from the digital process when compared to the conventional one. Both these methods end up with the same ratings of patient satisfaction with the final restorations. Digital workflow illustrated that the laboratory and total fabrication times got shorter and required less chairside adjustment in particular cases. Besides, the digital approach indicated a positive effect in terms of the patient-oriented factors, such as anxiety, high convenience, taste perception, nausea sensation, and pain. The study’s findings, however, not only indicate advantages for dental workflows digitally but also for esthetic outcomes, particularly if an IOS is used. Nevertheless, patient satisfaction-related aspects such as implant prosthetic function and esthetics may have variable results in both digital and conventional workflows. Hence, the consequences of this research may influence the work of dentists as well as the treatment of patients by introducing some considerations regarding cost efficiency, time efficiency, patient-focused outcomes, and esthetic results.

## References

[1] Schoenbaum TR (2012). Dentistry in the digital age: an update. Dent Today.

[2] Koch GK, Gallucci GO, Lee SJ (2016). Accuracy in the digital workflow: from data acquisition to the digitally milled cast. J Prosthet Dent.

[3] Haidar ZS (2023). Digital dentistry: past, present, and future. IntechOpen.

[4] Christensen GJ (2009). Impressions are changing: deciding on conventional, digital or digital plus in-office milling. J Am Dent Assoc.

[5] Beuer F, Schweiger J, Edelhoff D (2008). Digital dentistry: an overview of recent developments for CAD/CAM generated restorations. Br Dent J.

[6] Saeed EA, Alaghbari SS, Lin N (2023). The impact of digitization and conventional techniques on the fit of fixed partial dentures FPDs: systematic review and meta-analysis. BMC Oral Health.

[7] Gedrimiene A, Adaskevicius R, Rutkunas V (2019). Accuracy of digital and conventional dental implant impressions for fixed partial dentures: a comparative clinical study. J Adv Prosthodont.

[8] Bessadet M, Drancourt N, El Osta N (2024). Time efficiency and cost analysis between digital and conventional workflows for the fabrication of fixed dental prostheses: a systematic review. J Prosthet Dent.

[9] Page MJ, McKenzie JE, Bossuyt PM (2021). The PRISMA 2020 statement: an updated guideline for reporting systematic reviews. BMJ.

[10] Sakornwimon N, Leevailoj C (2017). Clinical marginal fit of zirconia crowns and patients' preferences for impression techniques using intraoral digital scanner versus polyvinyl siloxane material. J Prosthet Dent.

[11] Mangano F, Veronesi G (2018). Digital versus analog procedures for the prosthetic restoration of single implants: a randomized controlled trial with 1 year of follow-up. Biomed Res Int.

[12] Sailer I, Mühlemann S, Fehmer V, Hämmerle CH, Benic GI (2019). Randomized controlled clinical trial of digital and conventional workflows for the fabrication of zirconia-ceramic fixed partial dentures. Part I: time efficiency of complete-arch digital scans versus conventional impressions. J Prosthet Dent.

[13] Rattanapanich P, Aunmeungtong W, Chaijareenont P, Khongkhunthian P (2019). Comparative study between an immediate loading protocol using the digital workflow and a conventional protocol for dental implant treatment: a randomized clinical trial. J Clin Med.

[14] Mühlemann S, Benic GI, Fehmer V, Hämmerle CH, Sailer I (2019). Randomized controlled clinical trial of digital and conventional workflows for the fabrication of zirconia-ceramic posterior fixed partial dentures. Part II: time efficiency of CAD-CAM versus conventional laboratory procedures. J Prosthet Dent.

[15] Pan S, Guo D, Zhou Y, Jung RE, Hämmerle CH, Mühlemann S (2019). Time efficiency and quality of outcomes in a model-free digital workflow using digital impression immediately after implant placement: a double-blind self-controlled clinical trial. Clin Oral Implants Res.

[16] Zhang Y, Tian J, Wei D, Di P, Lin Y (2019). Quantitative clinical adjustment analysis of posterior single implant crown in a chairside digital workflow: a randomized controlled trial. Clin Oral Implants Res.

[17] Kunavisarut C, Jarangkul W, Pornprasertsuk-Damrongsri S, Joda T (2022). Patient-reported outcome measures (PROMs) comparing digital and conventional workflows for treatment with posterior single-unit implant restorations: a randomized controlled trial. J Dent.

[18] Capparé P, Ferrini F, Ruscica C, Pantaleo G, Tetè G, Gherlone EF (2021). Digital versus traditional workflow for immediate loading in single-implant restoration: a randomized clinical trial. Biology (Basel).

[19] Cheng CW, Ye SY, Chien CH, Chen CJ, Papaspyridakos P, Ko CC (2021). Randomized clinical trial of a conventional and a digital workflow for the fabrication of interim crowns: an evaluation of treatment efficiency, fit, and the effect of clinician experience. J Prosthet Dent.

[20] Ren S, Jiang X, Lin Y, Di P (2022). Crown accuracy and time efficiency of cement-retained implant-supported restorations in a complete digital workflow: a randomized control trial. J Prosthodont.

[21] Seth C, Bawa A, Gotfredsen K (2024). Digital versus conventional prosthetic workflow for dental students providing implant-supported single crowns: a randomized crossover study. J Prosthet Dent.

[22] Joda T, Brägger U (2015). Digital vs. conventional implant prosthetic workflows: a cost/time analysis. Clin Oral Implants Res.

[23] Joda T, Katsoulis J, Brägger U (2016). Clinical fitting and adjustment time for implant-supported crowns comparing digital and conventional workflows. Clin Implant Dent Relat Res.

[24] Ferrini F, Capparé P, Vinci R, Gherlone EF, Sannino G (2018). Digital versus traditional workflow for posterior maxillary rehabilitations supported by one straight and one tilted implant: a 3-year prospective comparative study. Biomed Res Int.

[25] Di Fiore A, Vigolo P, Graiff L, Stellini E (2018). Digital vs conventional workflow for screw-retained single-implant crowns: a comparison of key considerations. Int J Prosthodont.

[26] Yuce M, Ulusoy M, Turk AG (2019). Comparison of marginal and internal adaptation of heat-pressed and CAD/CAM porcelain laminate veneers and a 2-year follow-up. J Prosthodont.

[27] Delize V, Bouhy A, Lambert F, Lamy M (2019). Intrasubject comparison of digital vs. conventional workflow for screw-retained single-implant crowns: Prosthodontic and patient-centered outcomes. Clin Oral Implants Res.

[28] De Angelis P, Manicone PF, De Angelis S (2020). Patient and operator centered outcomes in implant dentistry: comparison between fully digital and conventional workflow for single crown and three-unit fixed-bridge. Materials (Basel).

[29] Hashemi AM, Hashemi HM, Siadat H, Shamshiri A, Afrashtehfar KI, Alikhasi M (2022). Fully digital versus conventional workflows for fabricating posterior three-unit implant-supported reconstructions: a prospective crossover clinical trial. Int J Environ Res Public Health.

[30] Edinger D, Henningsen A, Bibiza E, Smeets R, Joda T (2023). Comparison of functional and esthetic outcomes in digital versus analog rehabilitation of one-piece screw-retained implant crowns placed at second stage surgery. J Prosthodont.

[31] Sanchez-Lara A, Hosney S, Lampraki E (2023). Evaluation of marginal and internal fit of single crowns manufactured with an analog workflow and three CAD-CAM systems: a prospective clinical study. J Prosthodont.

[32] (2024). Study Quality Assessment Tools. https://www.nhlbi.nih.gov/health-topics/study-quality-assessment-tools.

